# A modeling and simulation study of siderophore mediated antagonism in dual-species biofilms

**DOI:** 10.1186/1742-4682-6-30

**Published:** 2009-12-22

**Authors:** Hermann J Eberl, Shannon Collinson

**Affiliations:** 1 Department of Mathematics and Statistics, University of Guelph, Guelph, On, Canada, N1G 2W1; 2 Department of Mathematics and Statistics, York University, Toronto, On, Canada, M3J 1P3

## Abstract

**Background:**

Several bacterial species possess chelation mechanisms that allow them to scavenge iron from the environment under conditions of limitation. To this end they produce siderophores that bind the iron and make it available to the cells later on, while rendering it unavailable to other organisms. The phenomenon of siderophore mediated antagonism has been studied to some extent for suspended populations where it was found that the chelation ability provides a growth advantage over species that do not have this possibility. However, most bacteria live in biofilm communities. In particular *Pseudomonas fluorescens *and *Pseudomonas putida*, the species that have been used in most experimental studies of the phenomenon, are known to be prolific biofilm formers, but only very few experimental studies of iron chelation have been published to date for the biofilm setting. We address this question in the present study.

**Methods:**

Based on a previously introduced model of iron chelation and an existing model of biofilm growth we formulate a model for iron chelation and competition in dual species biofilms. This leads to a highly nonlinear system of partial differential equations which is studied in computer simulation experiments.

**Conclusions:**

(i) Siderophore production can give a growth advantage also in the biofilm setting, (ii) diffusion facilitates and emphasizes this growth advantage, (iii) the magnitude of the growth advantage can also depend on the initial inoculation of the substratum, (iv) a new mass transfer boundary condition was derived that allows to a priori control the expect the expected average thickness of the biofilm in terms of the model parameters.

## Background

With but few exceptions, iron is absolutely required for life of all forms, including bacteria. It plays an important role in many biological processes, such as methanogenesis, respiration, oxygen transport, gene regulation and DNA biosynthesis [1]. Iron is abundant in the Earth. However, while in the early ages of life the predominant form of iron was rather soluble, it is now extremely insoluble and, therefore, the bioavailability of this minor nutrient is often low. To overcome iron limitations, some bacteria secrete iron-chelation compounds (so-called siderophores) when the environmental iron concentration becomes small. These bind with iron to form a siderophore-iron complex, which is then taken up by the cells and the iron is later liberated internally. This enables the microorganisms to scavenge iron from the environment which, thus, becomes unavailable to other organisms, including hosts.

Under iron limitations, species that produce siderophores and, thus, chelate iron can have a competitive advantage over species that lack this ability [2]. Such siderophore mediated antagonism has been observed in agricultural microbiology [3-5] and in food microbiology for some food spoilage bacteria, e.g. in meat, fish, poultry and dairy [6-10]. In these environments nutrients are often available in abundance, while iron can become growth limiting. The siderophore mediated antagonism is inversely related to the availability of iron [4] in the medium (soil or food); it is not observed if and when iron is not limited [2]. The bacteria that most experimental studies of this phenomenon focus on are pseudomonads, primarily of the *Pseudomonas fluorescens - P. putida *group, which produce a yellow-green (under UV light) pigment with high iron binding constant. This is the siderophore pyoverdine.

In the present study we focus on the antagonistic effect against other bacteria, as studied experimentally in [2], but the principle of growth suppression of other microorganisms by iron scavenging from the environment applies also to the control of yeasts; in a medical context this phenomenon has also been suggested as a mechanism to control cancer and other diseases. Because of their antagonistic effect, it is now generally recognized that plant pathogens with this property, in fact, can even have plant growth promoting effect by controlling wilt disease or other root crop diseases. Therefore, such PGPR (*plant growth promoting rhizobacteria *[5]) have been used for soil inoculation to increase yields.

The majority of experimental studies of iron chelation, as well as the population level modeling studies of pyoverdine production and iron chelation so far have been carried out for suspended cultures. Most bacteria, however, live in biofilm communities and not in suspended cultures. In particular the pseudomonads, which have been most commonly used in iron chelation studies are known to be natural and prolific biofilm formers. While there is increasing evidence that iron chelation can play an important role in biofilms [2,11-13], no conclusive quantitative studies of siderophore mediated antagonism in biofilms have been conducted so far. Previous laboratory studies of this question in [2] remained inconclusive, because of the affinity of one of the strains involved in the study towards the reactor material. Since the interaction of population and resource dynamics in biofilm communities can be very different from suspended cultures [14], it cannot be answered by straightforward inference from the planktonic case whether or not siderophore production provides a growth advantage. We approach this question by developing a mathematical model, which is then studied in computer simulations. Using a theoretical approach, it becomes possible to focus on the effect at the center of the investigation, without adverse perturbations to which laboratory studies are susceptible, like the ones reported in [2].

Bacterial biofilms are microbial depositions on surfaces and interfaces in aqueous systems. Biofilms form after individual cells attach to the surface, called substratum in the biofilm literature, and begin to produce extracellular polymeric substances (EPS), which form a gel-like layer in which the bacteria themselves are embedded. This polymeric layer offers protection against mechanical removal, but also against antimicrobials, that suspended bacteria do not have. One of the most striking differences between life in biofilms and in suspended cultures is that biofilm bacteria live in concentration gradients [14], due to decreased diffusion of dissolved substrates, the spatial organization of the cells, consumption and production of substrates, and biochemical reactions in the EPS matrix. This can lead to spatially structured populations with niches for specialists that cannot be found in suspension. For example, aerobic bacteria close to the biofilm/water interfaces can consume the oxygen in the environment and thus establish anaerobic zones in the deeper regions of the biofilm, closer to the substratum. Similarly, many antimicrobial agents only inactivate the bacteria closest to the biofilm/water interface but do not reach the cells in the deeper layer, which can survive an antibiotic attack virtually unharmed. In environmental systems biofilms are typically considered good, because their sorption and degradation properties contribute to soil and water remediation. Therefore, many environmental engineering technologies are based on biofilm processes, in particular in wastewater treatment, soil remediation, and groundwater protection. In industrial systems, biofilms are responsible for accelerated corrosion (microbially induced corrosion, biocorrosion) and biofouling. Biofilm contamination in food processing plants and hospitals are associated with public health risks [15-17]. In a medical context, biofilms can cause bacterial infections, which are diffiicult to treat with antibiotics, for the reasons indicated above. The list of biofilm originated diseases and infections is long and includes cystic fibrosis pneumonia, periodontitis and dental caries, and native valve endocarditis. A more detailed overview is given in [18]. In order to overcome the limitations of antibiotics in treating biofilm infections other strategies have been suggested recently, such as quorum sensing based methods [18,19], or iron chelation based methods [12].

Mathematical models for bacterial biofilms have been used for several decades and they have greatly contributed to our understanding of biofilm processes so far. The first generation of biofilm models were continuum models with a focus on population and resource dynamics, formulated under the assumption that a biofilm can be described as a homogeneous layer, cf [20]. In reality, however, biofilms can develop in rather irregular structures, such as cluster-and-channel architectures. Homogeneous biofilm layers are primarily obtained under conditions of abundance. Since we are interested in the iron chelation process, we are interested in situations of iron limitations. Therefore, a multi-dimensional biofilm model is required that supports the formation of cluster-and-channel biofilm architectures. In the past decade several such models have been developed [20,21]. The first group of these models, although utilizing a variety of different mathematical concepts, from individual stochastic based models to stochastic cellular automata, to deterministic continuum models, focused on biofilm growth, population and resource dynamics, i.e. on biofilm processes with typical time scales of days and weeks. This is what we need for our study. The second group of multi-dimensional models focuses on mechanical aspects of biofilms, such as biofilm deformation and eventual detachment, i.e. on processes on a much shorter time scale. Currently no biofilm model is known that connects both aspects reliably. Therefore, the latter processes are neglected in our model in the same manner as they are neglected in other biofilm growth models.

## Mathematical Model

We develop a mathematical model of siderophore production and iron chelation in biofilms by combining the iron chelation model [22,23], which was originally developed for batch cultures, with the density-dependent diffusion reaction model for biofilm formation that was originally introduced and studied for single-species biofilms, both for mathematical and biological interest, in [24-29] and extended to mixed-culture systems in [30-32]. Our focus here is on the growth advantage of siderophore producing bacteria over bacteria that lack this ability. Therefore, we formulate the biofilm model for a mixed culture biofilm. A related modeling and simulation study for suspended populations in batch and chemostat like environments was recently conducted in [33], where it was found with a blend of analytical and computational techniques, that iron chelation abilities can greatly affect persistence results in chemostats. Mathematical models of biofilms render the complexity of biofgilm populations. They are essentially more complicated than mathematical models of suspended microbial populations and most mathematical techniques than can be used to study suspended populations cannot be used to study biofilms. In particular, biofilm models do not lend themselves easily to analytical studies but must be investigated in time intensive computer simulations.

### Governing equations

Our biofilm formation model is formulated in terms of the dependent variables volume fraction occupied by the siderophore producing species, *N*, and volume fraction occupied by species that does not produce siderophores, *R*. We follow the usual approach of biofilm modeling and subsume the EPS that is produced by the bacteria in the biofilm volume fractions. The total volume fraction occupied by the biofilm is then *M *= *N *+ *R*.

In our modeling study we focus on siderophore mediated antagonism. Therefore, we assume that iron availability is the only growth limiting factor for the development of the biofilm; all other required nutrients are assumed to be available in abundance. Moreover, we assume that the growth conditions in the medium are not altered by the iron dynamics. Under iron limitations, the chelator produces the siderophore pyoverdine, denoted by *P*, which binds dissolved iron *S *and makes it unavailable to the non-chelator. This transformation from dissolved iron *S*, to chelated iron, *Q*, is assumed to be 1:1. The dissolved iron diffuses in the aqueous phase and, at a lower rate, in the biofilm. The species *R*, which does not produce the siderophore, requires dissolved iron, *S*, for growth, while the siderophore producer's growth is controlled by the total of available iron, dissolved and chelated, *S *+ *Q*. We assume that pyoverdine and chelated iron do not diffuse into the aqueous environment but are entrapped in the biofilm. The biofilm expands spatially, if the local cell density approaches the maximum cell density, i.e. if it fills up the available volume. It does not expand notably if locally space is available to accommodate new cells. This is described by a density-dependent diffusion mechanism, that shows two non-linear diffusion effects [25,34]: (i) it degenerates like the porous medium equation for vanishing biomass densities, and (ii) the diffusion coefficient blows up if the local cell density approaches its maximum value. Effect (i) causes the biofilm/water interface to spread at finite time, i.e., it guarantees a sharp interface between the biofilm and the surrounding aqueous phase. The super diffusion effect (ii) enforces volume filling, i.e. that the maximum cell density is never exceeded. Note that the interplay of both effects is necessary to describe biofilm growth.

**Figure 1:**
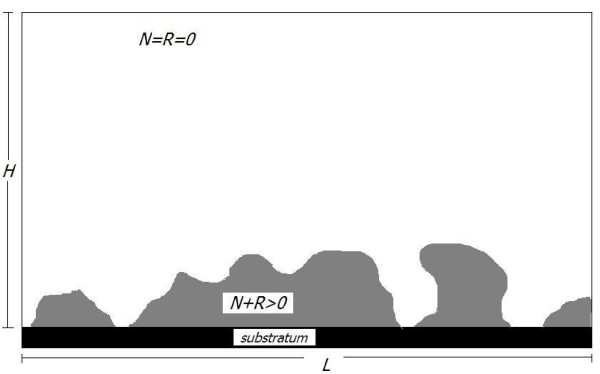
**Schematic of the computational biofilm system**: The computational domain Ω is assumed to be a rectangle of dimensions *L *× *H*. The actual biofilm is the area Ω_2_(*t*) = {*x *: *N *(*t*, *x*) + *R*(*t*, *x*) > 0}, surrounded by the aqueous phase Ω_1_(*t*) = {*x*: *N *(*t*, *x*) = *R*(*t*, *x*) = 0}, spearated by the interface Γ_1_(*t*) (not explicitly plotted). The biofilm grows on the bottom boundary, which represents the substratum.

The mathematical model for iron chelation and iron competiton in a dual species biofilm reads(1)

where as above(2)

is the total volume fraction occupied by the biofilm. We assume here that the volume fraction occupied by pyoverdine and the chelated iron is negligible compared to the volume fraction occupied by the bacteria and EPS. The biofilm is the region Ω_2_(*t*) = {*x *∈ Ω: *M *(*t*, *x*) > 0}, while the complement Ω_1_(*t*) = {*x *∈ Ω: *M *(*t*, *x*) = 0} denotes the surrounding aqueous phase. Both regions are separated by the biofilm water interface Γ(*t*) = , cf also Figure 1.

The density dependent diffusion coefficient that describes biofilm expansion reads(3)

Since pyoverdine and chelated iron are associated with the biofilm matrix we assume them to move at the same diffusive rate as the biofilm.

The diffusion coefficient *d*(*M*) for dissolved iron depends on *M *as well, albeit in a non-critical way. We make a linear *ansatz *that interpolates between the values of diffusion of iron in water, *d*(0) and in a fully developed biofilm, *d*(1),(4)

Unlike (3), the diffusion coefficient of iron *d*(*M*) is bounded from below and above by given constants of the same order of magnitude. Thus, diffusion of iron is essentially Fickian. Biomass spreading is much slower than diffusion of dissolved substrates, [20], thus the biomass motility coefficient is several oders of magnitude smaller than the substrate diffusion coefficients, ϵ ≪ *d*_1_; see Table 1 for the values used in this study.

**Table 1:** Model parameters used in this study

parameter	symbol	value	unit
growth rate, chelator	*μ*_1_	12.3	*d*^-1^
growth rate, non-chelator	*μ*_2_	12.3	*d*^-1^
half-saturation concentration, chelator	*k*_1_	3.7	*μM*
half-saturation concentration, non-chelator	*k*_2_	3.7	*μM*
decay rate, chelator	*d*_1_	0.49	*d*^-1^
decay rate, non-chelator	*d*_2_	0.49	*d*^-1^
yield coefficient, chelator	*Y*_1_	0.6003	-
yield coefficient, non-chelator	*Y*_2_	0.6003	-
maximum biomass density, chelator	*N*_∞_	10^4^	*gm*^-3^
maximum biomass density, non-chelator	*R*_∞_	10^4^	*gm*^-3^

chelation rate	*β*	1.92	
pyoverdine production rate	δ	2.56	*OD*_*P*_*d*^-1^
pyoverdine inhibition concentration	*S∞*	0.3762	*μM*
pyoverdine inhibition exponent	*n*	3	-

biomass motility parameter	ϵ	10^-12^	*m*^2^*d*^-1^
biomass interface exponent	*a*	4	-
biomass threshold exponent	*b*	4	-
diffusion coefficent of iron in water	*d*(0)	8.64·10^-4^	*m*^2^*d*^-1^
diffusion coefficent of iron in biofilm	*d*(1)	7.776·10^-4^	*m*^2^*d*^-1^

The iron chelation reaction terms in the biofilm model (1) are a slightly generalized from those that have been proposed and identified for the suspended batch culture population model in [22]. In the latter the saturation that is described by Monod kinetics is not relevant for practical purposes since always *Q *≪ *k*_1 _and *S *≪ *k*_1 _after a very short initial transient phase. Therefore, first order reactions could be assumed.

This is not necessarily the case in the biofilm setting, depending on the amount of iron supplied to the system, where the iron concentration can be very different between locations close to the substratum and at the biofilm/water interface. Therefore, an extension of the model to Monod kinetics became necessary. Analytical results for density-dependent diffusion-reaction models with degeneracy and super diffusion effects as implied by (3) can be found in [24,27-29,32,35]. These include existence results and for single-species models also uniqueness results, as well as studies on long term behaviour and stability. The study [34] gives a derivation of this deterministic, fully continuous model from a discrete-space model that is based on local behavioural rules similar to cellular automata models for biofilm growth, e.g [36-38]. Moreover, the underlying prototype biofilm model [25] can also be derived with hydrodynamic arguments similar to those used in [39] but under weaker assumptions, (cf Frederick et al, "A mathematical model of quorum sensing in patchy biofilm communities with slow background flow", submitted). Biological systems that have been previously described using this modeling approach include disinfection of biofilms with antibiotics [26,35], competition between species for shared substrates [30,40,41], and amensalistic control [31,32,42].

### Initial and boundary conditions

In order to close model (1) above, suitable initial and boundary conditions must be specified.

#### Initial conditions

In laboratory experiments the inoculation of the substratum, i.e. the sites at which the cells initially attach to the substratum, is difficult to control and appears random. In most of our simulations (except where noted) below, we will mimic this by choosing the actual sites of attachment at the substratum randomly. However, in order to ensure comparability across simulations we specify the initial number of colonies of both bacterial species as input data. Moreover, the volume fraction occupied by biomass in these inoculation sites is chosen randomly (uniform) between a given minimum and maximum value.

The initial biomass densities *N *and *R *are thus positive in the attachment sites on the substratum and 0 everywhere else. Initially, we choose a constant dissolved iron concentration *S*(0, ·) = *S*_0 _= 2 *μM *below the half saturation concentrations *k*_1 _and *k*_2 _but higher than the pyoverdine inhibition concentration *S*_∞ _that triggers the chelation process. Both, the concentration of chelated iron *Q *and the pyoverdine concentration *P*, are assumed to be 0 initially.

While (1) represents a completely deterministic model, this choice of inoculation adds a stochastic element.

It is naturally expected that different inoculation sites lead to different local biofilm morphologies and, hence, to different substrate distributions, but it is not clear *a priori *whether this also affects global, lumped results such as bacterial population sizes, mass conversion rates etc. For example, in [31] an amensalistic biofilm control strategy was investigated where the actual initial distribution of the control agent relative to the pathogen determines success or failure of the control strategy. Other studies, such as [26,40] showed no or only little quantitative and no qualitative effect of inoculation sites on global measurements. The modeling studies of the impact of inoculation sites on biofilm processes conducted so far allow the conclusion that it depends on (i) the type of interaction between species (e.g., competition, amensalism), (ii) the response to limiting substrates (e.g., growth, disinfection), and (iii) density (vs. sparsity) of the inoculation. Since the effect of inoculation on the overall biofilm performance is *a priori *not clear, it is advisable to run simulation experiments in the form of trials with several replicas, cf also [40]. This is the approach that we take in this study.

#### Boundary conditions

A so far only unsatisfactorily solved, open problem in mesoscopic biofilm modeling is the specification of boundary conditions for the dependent variables. While it is relatively straightforward to prescribe boundary conditions for biomass and biomass associated components of the biofilm, formulating appropriate boundary conditions for dissolved, growth limiting substrates requires more thought.

**Figure 2:**
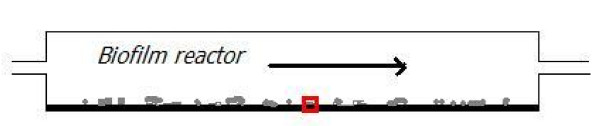
**Schematic of a flow-through biofilm reactor**. The computational domain Ω is depicted in this reactor as a red box.

The problem stems from the fact that due to computational limitations in numerical experiments only a small section Ω of an entire biofilm reactor can be simulated, cf Figure 2. While it is straightforward to describe boundary conditions for the reactor as a physically closed system, this is more difficult and not straightforward for Ω as a subsystem with open physical boundaries. Here the boundary conditions connect the computational domain with the outside world, i.e. need to reflect the external physical process that have an effect on the processes inside the computational domain.

For biofilm and biofilm matrix associated components, in our case biomass fractions, chelated iron and pyoverdine, traditionally no-flux conditions are assumed,(5)

where ∂_*n *_denotes the outer normal derivative at the boundary of the domain. This ensures that no biomass or biomass associated components leave or enter the domain across the boundary. For the part of the boundary of the domain that consists of the substratum this is the natural boundary condition. For the lateral boundaries these are symmetry conditions, which enable us to view the small simulation section as a part of a much larger system.

More problematic is the formulation of boundary conditions for the growth promotong substrate *S*. It is easily verified that a no-flux condition, such as (5), everywhere at the boundary of the computational domain will not allow for a biofilm to form. Under these conditions the bacteria can only utilize the iron that is initially in the system. Integrating (1) over Ω, and adding the equations for *N*, *R*, *S*, *Q *we obtain with ∂_*n *_= 0 and the Divergence Theorem that

That *N *and *R *are indeed non-negative follows with arguments that have been worked out in [29,32]. This implies that the total amount of biomass in the system is bounded by the initial amount of iron and biomass in the system and that biomass is in fact eventually decreasing. More specifically we have

In other words, in order to obtain enough biomass for a noteworthy biofilm community, the domain Ω must be huge relative to the desired biofilm size. Otherwise, all iron will be immediately consumed before a biofilm can develop. Hence, since for computational reasons the domain size Ω must be restricted, the boundary conditions must include a mechanism that describes replenishment of the consumed substrate, even if it is expected to become limited eventually.

Usually this problem is dealt with by prescribing the concentration of the dissolved growth promoting substrate on some part of the boundary (Dirichlet condition), often the boundary opposing the substratum, while no-flux conditions are specified everywhere else, which can be interpreted in the same manner as above for the biomass associated components. When the biofilm grows, the substrate concentration inside the domain decreases due to consumption. However, since under these boundary conditions the concentration is fixed along the Dirichlet boundary, this leads to an increased substrate gradient into the domain there, and, thus, to an increasing diffusive flux into the domain as the biofilm grows. Hence, if Dirichlet conditions are specified to model substrate replenishment, biofilm growth implies an increased supply of growth limiting substrate. Since we are here interested in studying biofilms under substrate limitations, which trigger the chelation mechanism, this is not appropriate for our application. In order to alleviate the effect of increasing substrate supply in response to biofilm growth we propose here two alternative boundary conditions to describe substrate replenishment.

##### Iron boundary condition I

**Figure 3:**
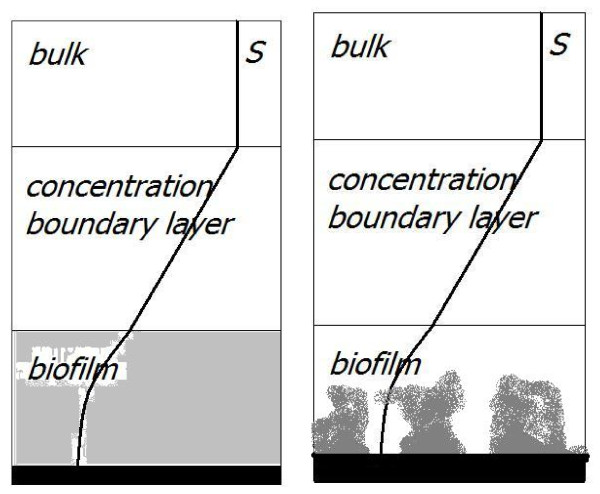
**Concentration boundary layer concept for boundary conditions**. Left: Traditional 1D model representation of a biofilm, consisting of the actual biofilm, the concentration boundary layer and the completely mixed bulk, cf. [20]. Right: Adaptation of this concept to multi-dimensional meso-scopic biofilm models.

We adapt an idea from traditional 1D biofilm modeling, commonly used with the classical Wanner-Gujer model, cf [20] and Figure 3. In these one-dimensional models the biofilm system is typically represented by three compartments: (i) the actual biofilm with thickness *L*_*f *_in which the dissolved substrates are transported by diffusion and depleted in reactions, (ii) the so called concentration boundary layer with thickness *L*_*BL *_in which dissolved substrates are transported by diffusion, and (iii) the bulk phase, in which the substrate is assumed to be completely mixed and constant, cf [20]. Across the biofilm/water interface the concentration and the diffusive flux are continuous. Moreover, it is customary in 1D biofilm modeling to invoke a quasi steady state assumption based on the observation that the characteristic time-scale of substrate diffusion and reaction is small compared to the characteristic time scale of biofilm growth [20]. Under this simplifying assumption, the iron concentration in a 1D system is described by the following two-point boundary value problem for the dependent variable *S*,

for 0 <*y *<*L*_*f *_and

at the substratum, *y *= 0, and the biofilm/water interface, *y *= *L*_*f*_. These boundary conditions have two parameters, the bulk concentration *S*_0 _and the thickness of the concentration boundary layer *L*_*BL*_, i.e., one parameter more than the traditional Dirichlet condition. Note that this concentration boundary layer is an abstract, not experimentally observable concept. It is qualitatively related to the bulk flow velocity, in the sense that a small bulk flow velocity implies a thick concentration boundary layer, while a thin concentration boundary layer represents fast bulk flow. However, a quantitative co-relation between these two quantities is yet unknown [20].

This concept of a concentration boundary layer can be straightforwardly adapted from one-dimensional biofilm modeling to biofilm models like (1) in the rectangular domain Ω = [0, *L*] × [0, *H*], cf. Figure 3. Then the boundary conditions for iron are(6)

Thus the boundary condition for iron is a mixed boundary condition consisting of a homogeneous Neumann boundary and a Robin boundary. Compared to the traditional Dirichlet boundary condition discussed above it has the effect that the growing biofilm not only lowers the substrate concentration inside the domain, but also on the boundary. While the diffusive flux into the system still increases with increasing biofilm size, it is bounded by *d*(0)*S*_0_/*L*_*BL*_. In the case of the Dirichlet condition, on the other hand, it grows unbounded. Thus iron replenishment will be slower under (6) than under the usually used Dirichlet conditions.

##### Iron boundary condition II

Increasing substrate supply as a consequence of a growing biofilm can be avoided, if the diffusive flux into the system is *a priori *fixed. This leads to a non-homogeneous Neumann condition on some part of the boundary. It reads(7)

where ∂Ω_*N *_denotes the part of the boundary of Ω on which the diffusive flux is prescribed, while its complement is the part on which no-flux conditions are specified. In order to relate the new parameter Σ to model parameters and biofilm properties, we consider, for simplicity, a single species biofilm that consists of the non-chelator only. Integrating the equation for *R *over Ω we have

Similarly, integrating the equation for *S *over Ω, using (7) and the Divergence Theorem yields

Invoking the same quasi-steady state argument as above, namely , these two equations can be combined to obtain the linear first order constant coefficient ordinary differential equation

for the total volume fraction occupied by biomass, ℛ:= ∫_Ω _*Rdx *It is easy to verify that for *t *→ ∞ the biomass volume fraction attains the asymptotically stable steady state

In other words, the boundary condition (7) allows us to specify a target size for the biofilm and to choose the boundary condition parameter Σ accordingly. A mathematically equivalent but more convenient measure for the biofilm than the total volume fraction occupied is the target biofilm thickness

where ∂Ω_*S *_denoted the part of the boundary of Ω that forms the substratum. The parameter λ is the average thickness that a completely compressed biofilm would have, i.e. a biofilm for which *R *≡ 1 in Ω_2_.

We recall that indeed many computer simulations of the underlying biofilm model have shown that in the interior of a growing biofilm *R *≈ 1, cf [25,29,43], while other biofilm models, such as [39] are based on the model assumption of an always completely compressed biofilm. Thus the model parameter Σ of the boundary condition (7) can be related to model parameters and the target biofilm thickness λ by(8)

If, as in our simulations and in the vast majority of biofilm modeling studies in general, Ω is rectangular, and if the substrate flux is applied on the opposite side of the substratum, then the integral terms in (8) cancel out.

When using this boundary condition we will specify it in terms of λ, rather than the actual substrate flux. Unlike the previous boundary condition (6) and the more traditional Dirichlet boundary condition discussed above, the non-homogeneous Neumann boundary condition allows us not only to estimate but to control the size that the biofilm will eventually have.

Note that (5) together with a boundary condition for *S*, such as (6) or (7) suffices. Since the solutions of the diffusion-reaction system (1) are understood in the weak sense, no internal boundary conditions must be specified across the biofilm/water interface to close the model, which, however, are necessary for other biofilm models, such as [44].

### Parameters

The model parameters used in this study are summarized in Table 1. In [22,23] a set of model parameters of the chelation process was determined from laboratory experiments with batch cultures of *Pseudomonas fluorescens*. In the absence of measurements for the biofilm setting, this is also what we use here. The remaining parameters for the biofilm growth model were chosen in the usual parameter range, cf. [20] and [25]. In order to ensure that competition effects are entirely due to differences in the strains' ability to utilize chelated iron, we choose that both species have the same specific growth rate *μ*_1 _= *μ*_2_, half saturation constant *k*_1 _= *k*_2_, yield coefficient *Y*_1 _= *Y*_2 _and decay rate *d*_1 _= *d*_2_. Thus, we assumed that *X*_2 _is a genetic modification of *X*_1_, which switches off iron chelation but leaves the growth kinetics unaffected.

### Computational realisation

The mathematical model (1) is discretized on a regular grid using an non-standard finite difference scheme for time integration and a second order finite difference based finite volume discretization. This is a straightforward adaptation of the method that has been introduced in [43] for single species biofilms and extended to mixed-culture systems in [31]. The main difference between (1) and other mixed-culture applications of the nonlinear diffusion-reaction biofilm model is that *P *and *Q *are controlled by the degenerate-singular diffusion operator, which, however, does not depend on *P *and *Q *directly. Thus, in the discretization these two equations behave essentially like semi-linear equations which to incorporate into the simulation algorithm does not pose any new problems. In every time-step, five sparse linear systems need to be solved, one for each dependent variable. This is the computationally most expensive part of the simulation code and was prepared for parallel execution on multi-processor/multi-core computers using OpenMP; cf [41] for a more detailed discussion of this aspects, where this approach was applied to a dual-species biofilm system that plays a role in groundwater protection. For the simulations presented here usually four threads were used on a SGI Altix 330 system. The visualisation of simulation results shown here were created using the Kitware ParaView visualisation package.

## Numerical experiments

### Simulations illustrate siderophore mediated growth advantage

**Figure 4:**
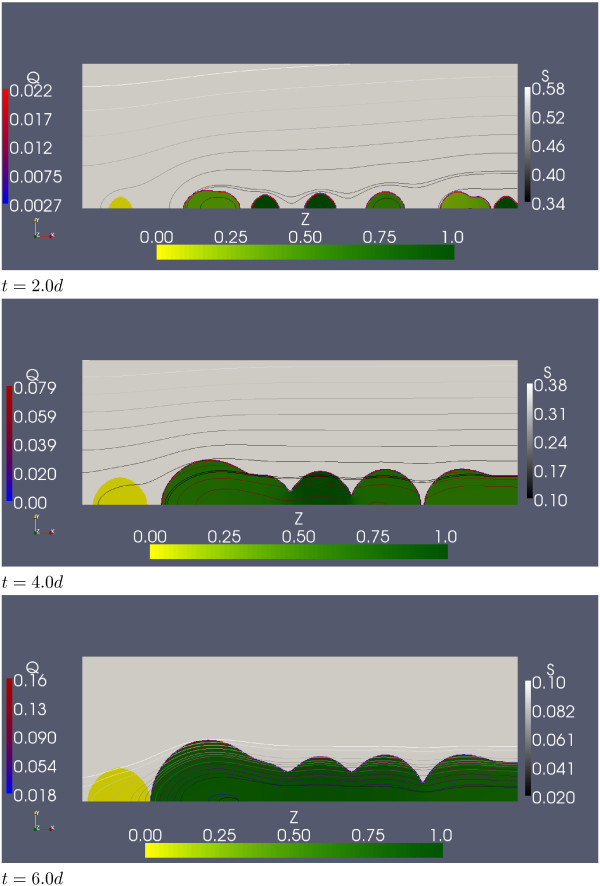
**Development of a dual-species biofilm formed by *N *and *R***. For selected time instances the biofilm morphology is depicted. The biofilm is coloured with respect to the fraction of the biofilm that is occupied by the chelator, *Z *:= *N/*(*N *+ *R*), using a yellow-green colour map. Also shown are iso-lines for the concentration of dissolved iron *S *in greyscale, and for chelated iron *Q *a blue-red color map.

**Figure 5:**
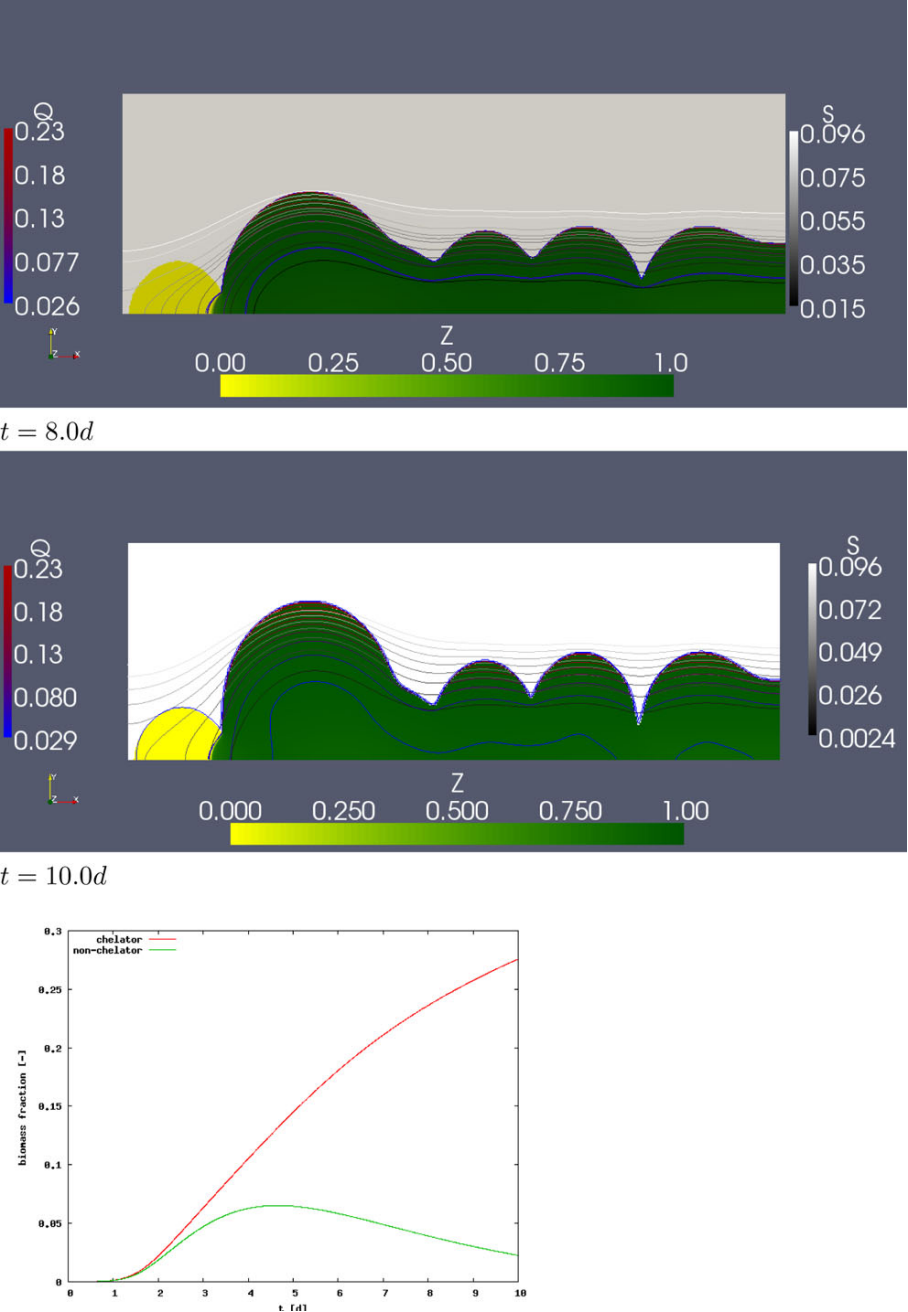
**Figure 4 continued**. The bottom insert shows the amount of the siderophore producer *N *and of the species that cannot produce pyoverdine, *R*, in the system as a function of time.

A typical simulation of model (1) is visualized in Figures 4 and 5. The computation was carried out on a grid with 600 × 200 cells and size *L *× *H *= 1.5 *mm *× 0.5 *mm*. Initially the substratum is inoculated in 6 randomly chosen sites each for the siderophore producing and the non-chelating species. The initial biomass volume fraction in these sites are randomly chosen between 0.2 and 0.4. Iron replenishment is in this simulation described by Robin boundary conditions (6) with concentration boundary layer thickness *L*_*BL *_= 1 *mm*.

In Figures 4 and 5 the biofilm morphology is shown for five selected time instances, together with iso-concentration lines for dissolved iron *S *and chelated iron *Q*. In order to show the relation between chelator and non-chelator, the biofilm region Ω_2_(*t*) is color-coded with respect to the variable

where *Z *= 0 (only non-chelators, no siderophore producers) is depicted in yellow and *Z *= 1 (only siderophore producers, no non-chelator) in dark green.

The biofilm grows throughout the simulation experiment, despite the maximum concentration of dissolved iron being clearly smaller than the half saturation constant, i.e. despite growth limitations. The simulation starts out from twelve small initial colonies. As these colonies grow bigger they grow closer together and eventually neighboring colonies merge into bigger colonies. At *t *= 2*d*, we observe three mixed-culture colonies, three clearly siderophore producer dominated colonies and one non-chelating colony. At *t *= 4*d *the non-chelating colony remains separated from the other colonies which now merge into two large mixed-culture colonies, which at *t *= 6*d *merge into one large clearly siderophore producer dominated mixed-culture colony. Also the non-chelating colony continues growing and the interfaces of the non-chelator and the mixed-culture colony collide at the substratum. For *t *= 8*d *and *t *= 10*d *we notice siderophore producers slowly invading the non-chelating colony. While the larger chelator dominated biofilm colony keeps growing toward the iron source, i.e. the top boundary, the non-chelator colony cannot grow further due to a severe limitation of dissolved iron *S*.

Initially, *S *took the bulk concentration value *S*_0 _= 2.0 *μM *but continuously decreases due to biofilm growth. By the end of the simulation the maximum concentration of dissolved iron in the system (attained at the upper boundary, where the replenished iron enters the system) drops to *S *≈ 0.23 *μM*. The iron concentration *S *is smaller in the chelator dominated colonies than in the non-chelator colony. In the larger chelator dominated biofilm colonies, the iron concentration *S *drops below *S*_∞ _and chelation starts. Thus, in addition to dissolved iron being directly consumed by chelators and non-chelators alike it is scavenged from the environment and transformed into chelated iron *Q *by the chelator. This leads to a diffusive flux of dissolved iron from the non-chelator colony into the chelator dominated colony. Hence iron does not only enter the mixed-culture colony from the top boundary but also laterally.

The chelated iron that accumulates in the biofilm increases over time. By the end of the simulation, the maximum concentration of chelated iron in the biofilm exceeds the maximum concentration of dissolved iron in the biofilm by a multiple. The chelated iron concentration is generally highest at the biofilm water interface, where also the concentration of dissolved iron is highest, and decreases toward the substratum. Since dissolved iron in the biofilm is limited, the continued growth of the mixed-culture colony is primarily due to chelated iron, i.e. the chelating population increases relative to the non-chelating population. In addition to the biofilm morphology and local quantities, we plot in Figure 5 also the amount of biomass of chelator and non-chelator in the system as a function of time and normalized by system size. These are computed as

Initially, up to *t *≈ 1*d*, as long as iron is not limited, both species grow at about the same rate. After that, the growth of the species that does not produce siderophores lags behind the siderophore producer's growth, indicating the expected growth advantage. Eventually, at about *t *≈ 4, the population that is not able to chelate declines, while the chelating population continues growing throughout the simulation, albeit at a decreased rate. The simulation stops at *t *= 10*d*, where, as indicated already before, all the non-chelator is accumulated in a single colony that is not yet notably invaded by the siderophore producer.

### Simulations with controlled inoculation show that the effect of siderophore mediated antagonism is sensitive to initial attachment sites

In order to investigate the effect of the competition between siderophore producing and non-producing species further we conduct a small simulation experiment, in which the initial biomass distribution is controlled in the following manner. Initially, the substratum is only inoculated by two colonies of identical, semi-spherical shape. One is situated at the left end of the simulation domain and one at the right end of the simulation domain.

The simulations are carried out on a grid of 300 × 200 cells covering a computational domain of size *L *× *H *= 0.75 *mm *× 0.5 *mm*.

We differentiate between the following four cases

(a) Two simulations are conducted. In one of them, both colonies are siderophore producers with an initial biomass density *N*_0 _= 0.3 (*R*_0 _= 0.0). In the second simulation both colonies are formed by the non-chelating species, *R*_0 _= 0.3 (*N*_0 _= 0). The concentration boundary layer thickness is set at *L*_*BL *_= 500 *μm*.

(b) The same as (a) but with a thicker concentration boundary layer *L*_*BL *_= 1000 *μm*, implying reduced rate of iron replenishment.

(c) A simulation in which one of the colonies is a single-species siderophore producer colony with initial biomass volume fraction *N*_0 _= 0.3, *R*_0 _= 0, the other colony is a single-species colony that is not able to produce siderophores, with *R*_0 _= 0.3 and *N*_0 _= 0. The concentration boundary layer is as in (b), *L*_*BL *_= 1000 *μm*.

(d) A simulation in which both colonies are identical, occupied by equal parts of each species, *N*_0 _= *R*_0 _= 0.15. The concentration boundary layer is as in (b), *L*_*BL *_= 1000 *μm*.

Thus, in all four scenarios the total amount of biomass, and, hence, iron consumers is initially the same, which enables comparison across simulations.

**Figure 6:**
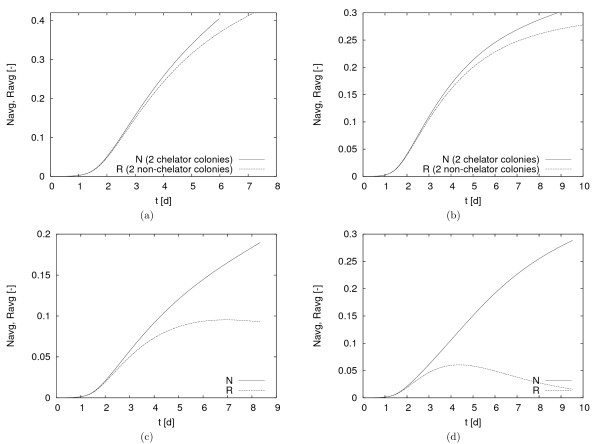
**Total amount of chelator and non-chelator in the system**. (a) both colonies of the same kind, *L*_*BL *_= 500 *μm *(b) both colonies of the same kind, *L*_*BL *_= 1000 *μm *(c) two competing species: one chelator, one non-chelator colony, *L*_*BL *_= 1000 *μm *(d) two mixed colonies, *L*_*BL *_= 1000 *μm*.

In all simulations the two colonies remained separated throughout, i.e. did not merge (simulations not shown). The amount of both species, *N*_*avg *_(*t*) and *R*_*avg*_(*t*), for these four cases are plotted in Figure 6. In the non-competition cases (a) and (b) we observe that initially, up to *t *≈ 2, both species indeed grow equally fast but eventually the non-chelating species grows slower than the siderophore producer, even in the absence of the direct competitor. This is explained by Fickian diffusion as the mechanism that supplies the colonies with growth controlling substrate *S*. The chelation mechanism, by converting *S *into *Q*, acts as a sink for dissolved iron *S *in the biofilm colony. This accelerates diffusion of iron into the colony and thus leads to a faster supply of iron to the actual biofilm. The Robin boundary conditions are such that the bulk iron concentration *S*_0 _is fixed. Therefore, higher iron demand, i.e. lower iron concentrations, imply a steeper iron gradient into the computational domain, and thus a higher iron supply. Therefore, in the case of these boundary conditions, more iron is supplied in the simulations of the siderophore producer *N *than in the simulation of the species *R *that lacks this ability. Comparing cases (a) and (b) directly, we notice, as expected, that the colonies grow faster in the case of faster iron replenishment, i.e. in case (a) where iron is less severely limited.

In the competition case (c), where two separate single species colonies are considered, we observe again that initially, as long as iron is not severely limited both colonies grow at the same rate. Eventually the growth of *R *lags behind the growth of *N*. At *t *≈ 7*d *the amount of *R *starts decreasing as a consequence of iron limitation, while the siderophore producer colony keeps growing, albeit at a decreased rate. The competition between both colonies in this case is non-local in the sense that both species are spatially separated. Fickian diffusion of dissolved iron is the facilitator of this competition. As discussed in the context of cases (a) and (b), the chelation mechanism is a sink for dissolved iron. Therefore, the iron concentration *S *in the chelating colony is lower than in the non-chelating colony (while the concentration of chelated iron is higher). This leads to a diffusive flux from the non-chelator to the chelator. The iron gained by the chelator in this way is converted into chelated iron and utilized for growth. Eventually the iron available to the non-chelator drops below the levels that are required to sustain growth.

In the competition case (d), where two mixed culture colonies are considered, the overall picture is qualitatively similar as in case (c), however with big quantitative differences. The maximum amount of biomass that is reached by the non-siderophore producing species *R *remains below the one of case (c) and is attained earlier. On the other hand the chelator *N *grows faster and to larger population levels. The competition for iron between both species is direct and local. Both have access to the same amount of dissolved iron, however, the chelating species scavenges some or most of it and transforms it into chelated iron that cannot be utilized by its competitor, which, therefore, eventually stays behind in its development and is out-competed.

In summary, the simulations of cases (a) - (d) not only clearly show the absolute growth advantage of the chelator compared to the non-chelator but also indicate that the competition can be due to two different effects, namely direct competition for dissolved iron and the advantage that the chelator gains by scavenging, as well as indirect competition that is facilitated by mass transfer of the growth controlling substrate, dissolved iron *S*, from regions, in which no chelation takes place, to regions, in which chelation takes place. However, the simulations imply, by comparing (c) with (d), that local competition is stronger than the non-local diffusion facilitated competition.

Moreover, this simulation experiment indicates that the overall competition for dissolved iron depends quantitatively on the initial distribution of chelator and non-chelators relative to each other. In mixed-species colonies competition is direct and fiercer than between separated single species colonies. Initial attachment sites are difficult to control in biofilm experiments and appear to some extent stochastic. In order to investigate the effect that this random inoculation of both species has on the iron chelation process, we conduct the following simulation experiment.

### Simulations with uncontrolled inoculation show that sensitivity to initial attachment sites is due to substrate diffusion

We conduct two simulation experiments, one for each of the iron replenishment mechanisms described above. One simulation trial consists of ten biofilm growth simulation with different randomly chosen biomass inoculations, where across the trial the number of inoculations sites is kept constant and also the minimum and maximum values between the volume fractions in the inoculation sites are chosen constant across the simulations in one trial.

#### Boundary layer concentration prescribed (Robin conditions)

The computations were carried out on a grid with 600 × 200 cells and size *L *× *H *= 1.5 *mm *× 0.5 *mm*. Initially the substratum is inoculated in 6 randomly chosen sites each for the chelator *N *and the non-chelator *R*, with initial volume fraction in these sites randomly chosen between 0.2 and 0.4. Iron replenishment is in these simulation described by Robin boundary conditions with concentration boundary layer thickness *L*_*BL *_= 1 *mm*.

**Figure 7:**
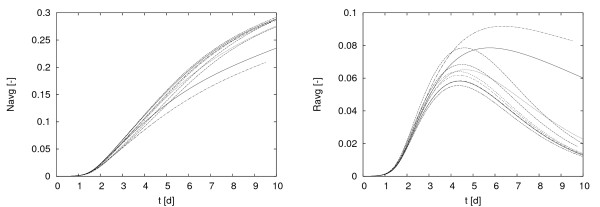
**Simulations of model (1) with different initial biomass distributions**. Siderophore producers, *N*_*avg *_(*t*) are shown in the left panel, non-siderophore producers, *R*_*avg *_(*t*), right. This simulation uses Robin boundary conditions for iron replenishment with concentration boundary layer thickness *L*_*BL *_= 1000 *μm*.

Figure 7 shows *N*_*avg *_(*t*) and *R*_*avg *_(*t*) for all ten simulations in the trial. The chelating population is continuously growing while the non-chelating population increases first and decreases eventually. It passes through its maximum between *t *≈ 4 and *t *≈ 6. Generally, the maximum population size that is attained by the non-chelating population remains below the population size that is achieved by the chelating population. However, before this maximum is achieved both populations develop at approximately the same rate, which indicates that the iron concentration is high enough to not trigger chelation. After chelation starts, the siderophore producers have a clear growth advantage.

The specific biofilm morphology that develops is of course clearly affected by the initial inoculation sites. The population sizes themselves show the same qualitative behavior for all simulations in the trial. However, there are quantitative differences. For example, in addition to the difference in the time at which *R*_*avg *_starts declining there are also differences observed in the maximum population size, ranging between *R*_*avg *_≈ 0.05 and *R*_*avg *_(*t*) ≈ 0.09, i.e. by a factor of 80%. A trend seems to be that the population at maximum is the higher, the later it is achieved, although there are some exceptions. The simulations were stopped at *t *= 10*d*, at which time the chelator population size varied between *N*_*avg *_= 0.2 and *N*_*avg *_= 0.3, i.e. by a factor of 50%. This suggests that the initial inoculation sites can affect the outcome of the simulation experiment quantitatively.

#### Iron flux prescribed (nonhomegenous Neumann conditions)

The boundary conditions (7) are designed to grow a biofilm with an a priori specified average biofilm thickness in the sense defined above. The previous simulation experiments have shown rather high variations in biofilm size as a consequence of variations in substratum inoculation. Furthermore, as discussed above, the substrate flux into the domain increases when the substrate concentration in the domain decreases. The chelation process is an iron sink and thus increases substrate supply to the system under this boundary condition. The non-homogenous Neumann boundary condition (7) instead of (6) avoids this diffusion effect on the competition for iron, because the substrate flux is prescribed as a constant and independent of the biofilm and the substrate utilization itself. The anticipated biofilm size is prescribed as an input parameters of the boundary condition.

**Figure 8:**
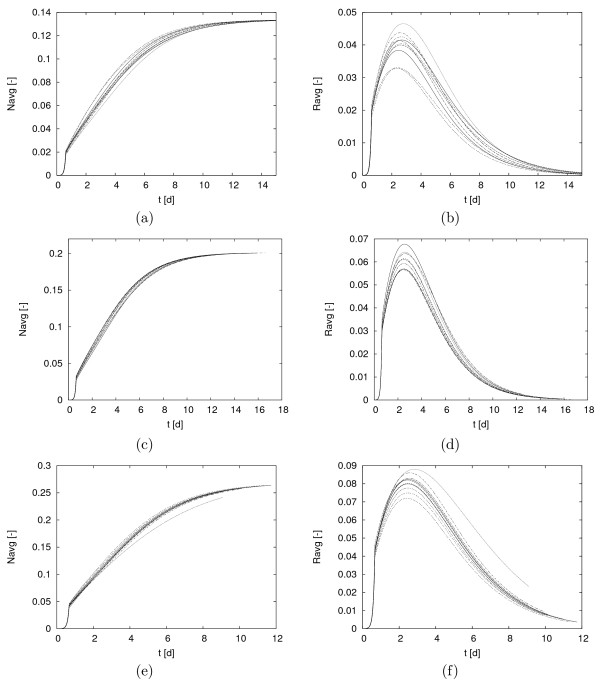
**Simulations of model (1) with different initial biomass distributions**. 10 simulations were carried out for three target biofilm thoicknesses *λ*. Shown is the amount of biomass in the system: (a) siderophore producer *N*, *λ *= 100 *μm*, (b) non-siderophor producer *R*, *λ *= 100 *μm*, (c) siderophore producer *N*, *λ *= 150 *μm*, (f) non-siderophore producer *R*, *λ *= 150 *μm*, (e) siderophore producer *N*, *λ *= 200 *μm*, (f) non-siderophore producer *R*, *λ *= 200 *μm*. Shown are the results of 10 simulations each.

Numerical experiments have been carried out for target biofilm thicknesses λ = 100 *μm*, 150 *μm*, 200 *μm*, using a grid of 400 × 300 cells to cover a physical domain Ω of size 1 *mm × *0.75 *mm*. Initially the substratum is inoculated with 6 randomly chosen pockets of siderophore producers and 6 randomly chosen pockets of bacteria that cannot produce siderophores. Again, 10 simulations were conducted in every trial. *N*_*avg*_and *R*_*avg *_are plotted in Figure 8. In all simulations one observes a transient initial period of rapid growth, which is neither observed in the cases of Robin boundary conditions (6) nor in the familiar case of Dirichlet conditions, in which the flux of iron into the system is initially small because of the low iron consumption of an initially very small bacterial population. In the case of the non-homogeneous Neumann boundary conditions (7), on the other hand the amount of iron supplied to the system does not depend on the current biofilm size. Therefore, in the initial period with a very small bacterial population, the substrate is not limited, leading to unhindered rapid growth and actually an iron accumulation in the environment. More iron is supplied than can be consumed by the still small bacterial population.

Eventually, after the biofilm has grown to a certain size and after the excess iron is depleted, the growth slows down and is only controlled by iron replenishment. The duration of this initial transient phase does not depend on the inoculation sites. The overall qualitative behavior with respect to population growth is in all cases as in the previous simulations: the species that cannot produce siderophores first grows but eventually declines and dies out, while the siderophore producing species survives. Since the maximum sustainable biofilm size is specified as an input parameter, in all simulations in one trial the population size converges to the same value, namely λ/*H*, where it reaches a plateau. In the transient phase between the initial growth period and the plateau phase, variations of population counts are observed across trials. The maximum populations sizes of the non-chelator vary between approximately 0.033 and 0.047 [λ = 100 *μm*; 42% variation], between approximately 0.055 and 0.068 [λ = 150 *μ*; 24% variation] and between approximately 0.070 and 0.088 [λ = 200 *μm*; 25% variation]. While inoculation induced variations across trials were observed in all cases, they seem smaller than in the case of the Robin boundary condition above. Similarly, the variations across population size of the chelator in one trial appear much smaller than in the simulation experiments in which the concentration boundary layer was prescribed instead of the diffusive flux. These observations indicate that in addition to a priori and explicitly controlling the size of the biofilm that is expected, using the non-homogeneous Neumann boundary conditions leads to population counts that are less sensitive to variations in the substratum inoculation.

**Figure 9:**
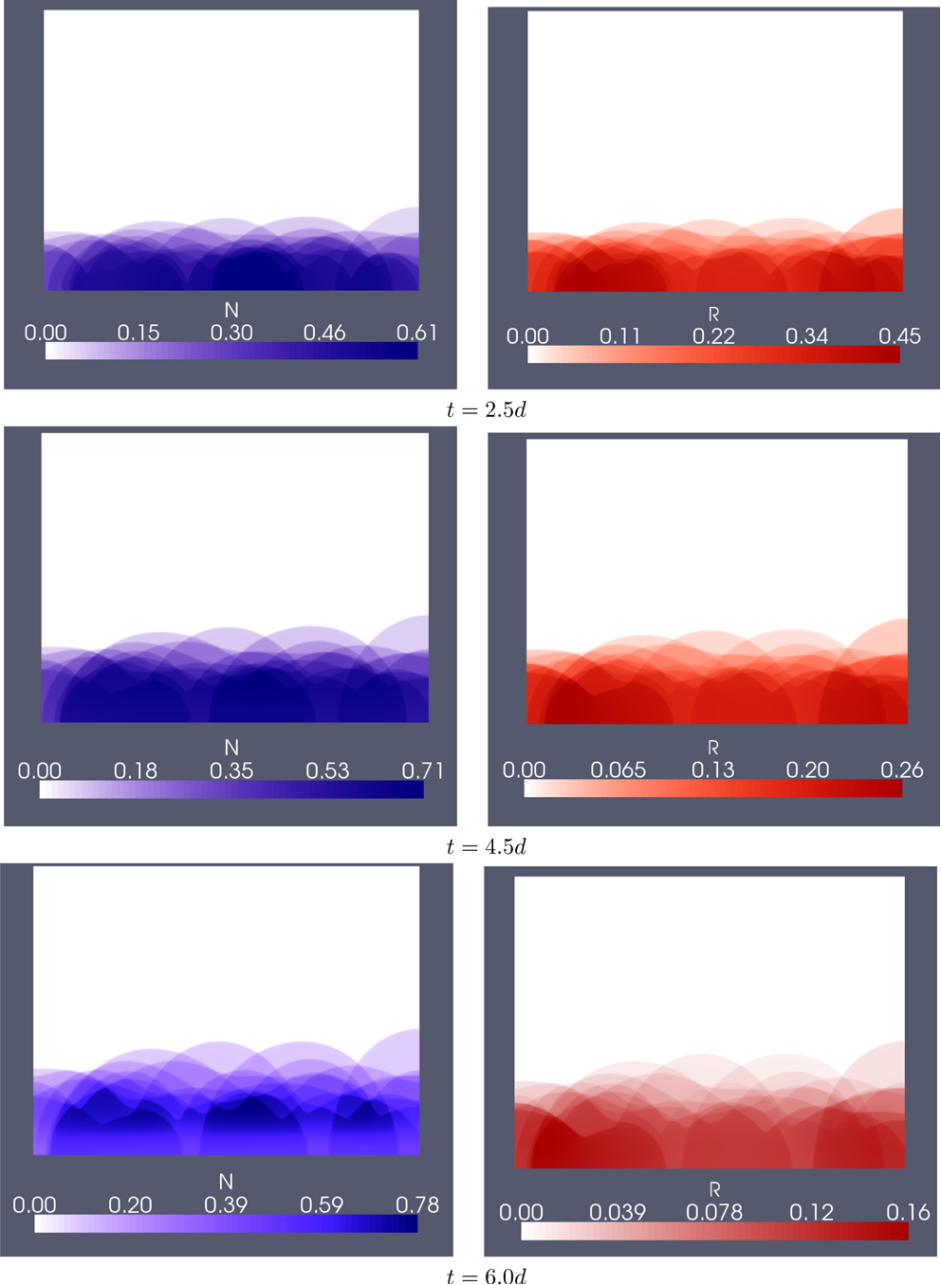
**Superposition of simulations**. Siderophore producers (left column) and non-producers (right column) averaged over 10 simulations with target biofilm thickness *λ *= 150 *μm *for three selected time instances.

**Figure 10:**
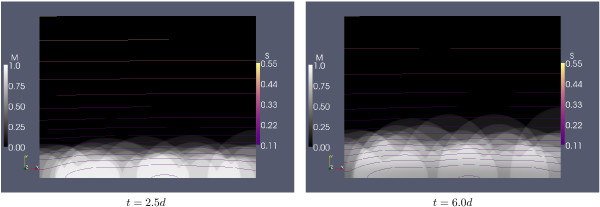
**Superposition of simulations**. Volume fraction occupied by biomass, *M *= *N *+ *R*, and iso-concentration lines for dissolved iron *S *for the simulations depicted in Figure 9. Iron concentrations are equidistantly spaced on a logarithmic scale.

In Figures 9 and 10 we superimpose the simulation results of the 10 simulations of the trial with λ = 150 *μm*. Shown are in Figure 9, for three selected time steps, the spatial distribution of the chelator and the of the non-chelator. The coarse structures of the spatial distribution of *N *and *R *are similar, but the differences in details indicate that the ratio of chelators to non-chelators differs between the individual colonies, as was already observed in the simulation of Figure 4 which was started from a sparser inoculation than the simulations in Figure 9. In Figure 10 we show the biofilm structure, in terms of volume fraction occupied by biomass *M *= *N *+ *R*, along with the concentration of dissolved iron *S *for *t *= 2.5*d *and *t *= 6.0*d*. The biomass fractions do not give the impression of a stratified biofilm after averaging but indicate cluster-and-channel biofilm geometries.

## Conclusions

We presented a mathematical model for siderophore mediated competitive advantage in dual-species biofilm systems under iron limitations. The model is based on previously developed building blocks, namely a density-dependent diffusion-reaction model for biofilm growth and a mathematical model for pyoverdine production and iron chelation. The model is studied in simulation experiments. So far most laboratory studies of iron chelation and siderophore mediated antagonism have been conducted with suspended cultures, while the biofilm setting has only received little attention. A notable exception is the study [2], in which, however, the siderophore mediated competition between *Pseudomonas fluorescens *and *Bacillus cereus *was also found to be influenced by the material of the substratum that was used in the experiments. Therefore, this study could not answer the question whether siderophore mediated anatagonism in biofilm communities is possible. While, for this lack of quantitative experimental data, our model predictions cannot be quantitatively validated against existing experiments, it allows us to draw the following conclusions.

1. Under iron limitations, siderophore producing bacteria have a growth advantage in biofilm systems, compared to bacteria that lack this ability. In contrast to the much better understood situation in suspended populations, this growth advantage is manifested in two aspects: (i) Direct competition between between bacteria that locally share the same space: The siderophores bind iron, which the siderophore producers can utilize later, while it becomes unavailable to their competitors. (ii) The chelation process is a local iron sink that induces Fickian diffusion toward siderphore producing colonies from colonies that do not produce siderophores. This increased supply of iron constitutes a further growth advantage for the siderophore producing species. Also in mixed colonies, siderophore producers benefit from Fickian diffusion of iron to the biofilm more than non-siderophore producers, because they convert the increased supply of dissolved iron to an increased supply of chelated iron. Comparing local, direct siderophore mediated antagonism (i) with the non-local mass transfer facilitated effects (ii), our results indicate that iron chelation provides a greater growth advantage in mixed-species colonies than between single species cultures of of siderophore producers and species that lack this ability. In suspended cultures, (i) is the only competition effect, while (ii) is specific to the biofilm setup. Indeed, in [14] it is argued that diffusion induced substrate concentration gradients is in many respects one of the major differences between life in biofilm communities and living in a suspended culture. Our results show that this applies to siderophore mediated antagonism under iron limitation as well.

2. The location where bacteria initially attach on the substratum are difficult to control or predict in experimental and natural biofilm systems. We mimicked this by stochastic inoculation. Our simulations indicate, however, that siderophore mediated antagonism and competition for iron can be greatly affected by the initial spatial distribution of biomass. Generally one expects that this sensitivity to attachment sites is smaller in densely inoculated systems (where one can expect that all colonies are soon mixed) than in sparsely inoculated systems (where single-species colonies can persist). The former is the case in soils and root systems where bacteria are found in abundance, while the latter is the case in food safety applications, where pathogens are hopefully scarce.

3. Boundary conditions for the dependent variables are an important part of every biofilm model. While formulating boundary conditions along physical boundaries is often unproblematic, the crux in biofilm modeling is that, due to computational limitations, all simulation studies are constrained to open sub-domain of physical systems. In this case, the boundary conditions connect the computational domain with the physical processes outside. In biofilm modeling the major issue is to describe the replenishment of consumed required resources through the boundaries of the computational domain. The boundary condition that is probably most frequently used to describe replenishment of consumed substrates is to prescribe the concentration value along some part of the boundary. Such Dirichlet conditions imply, as a consequence of diffusion of consumed substrates from the boundary toward the biofilm, that the amount of material that becomes available to the biofilm grows unbounded as the biofilm grows. This is not an appropriate description for modeling studies that focus on substrate limitations. We presented here two alternate boundary conditions that dampen or alleviate this effect. (i) The first one is a Robin boundary condition which introduces, in accordance with the boundary conditions used in traditional one-dimensional biofilm models, the abstract concentration boundary layer thickness *L*_*BL *_as an additional parameter. The traditional Dirichlet condition is a special case of this condition for *L*_*BL *_= 0. Introducing this concentration boundary layer puts an upper bound on the substrate flux in to the system but still implies that increasing biomass increases the flux into the system. (ii) The second boundary condition tested is a non-homogeneous Neumann condition. The substrate flux into the system can be simply correlated with the target biofilm size. Thus, this boundary conditions allows an easy control over the expected biomass accumulation in simulation experiments. Since the target biofilm size is a priori specified, the eventually prevailing siderophore producing species is less less sensitive to stochastic uncertainty effects in the longterm than in case of boundary conditions (i). Also the uncertainty effects in the growth curves of the non-siderophore producer, albeit still notable, are smaller than in case (i). The boundary conditions chosen not only affect simulation results quantitatively, but also qualitatively. Which boundary condition is more appropriate for a particular application depends on the reactor and the environmental conditions modeled. Therefore, sensitivity of the chelation process with respect to initial attachment can depend on the reactor type.

4. The model presented here is a bare-bone model that focuses solely on the competition effect. It can serve as a qualitative tool and guide experimental studies of the phenomenon. As it is the case with all multi-disciplinary research, this requires the collaboration of researchers with complimentary skill sets and research infrastructure. Such a cooperation between theoreticians and experimentalists will allow the experiments to feed back into and improve the theory. The mathematical model framework used here has been used to study several other biofilm systems before. It has been shown that this modeling concept as well as the computational techniques employed to study it are fairly flexible to incorporate additional biofilm processes from population and resource dynamics, such as competition for substrate, amensalism, biofilm response to biocides, convective transport of dissolved substrates, or quorum sensing. Therefore, the model presented here can serve as a starting point for model refinements and future improvements that may be revealed by experimental results to be necessary to account for a qualitatively and quantitatively accurate description of siderophore mediated microbial antagonism.

## Competing interests

The authors declare that they have no competing interests.

## Authors' contributions

HJE is the primary author of this contribution. MSC participated in this study as part of her M.Sc. program. Both authors read and approved the final manuscript.
